# American Dental Association

**Published:** 1886-11

**Authors:** 


### AMERICAN DENTAL ASSOCIATION.

TWENTY-SIXTH ANNUAL MEETING, HELD AT NIAGARA FALLS, AUG. 3, 4, 5, AND 6, 1886.

REPORTED FOR THE INDEPENDENT PRACTITIONER BY "MRS M. W. J."

#### WEDNESDAY EVENING SESSION.

The discussion of Operative Dentistry was continued for the exhibition and explanation of the appliances named in the report of Section IV, which was then passed.

Sections II and III were called, but were not ready.

Section V, Anatomy, Histology and Microscopy, reported through the chairman, Dr. Frank Abbott. He said that though little had been published during the year, much work of very great importance was being pushed forward, which would, in due time, be given to the profession. The only paper of especial interest in this line was one published in the INDEPENDENT PRACTITIONER, from Heitzman and Bödecker, entitled "Eburnitis, or Inflammation of Dentine." Dr. Abbott reminded the Association that, in 1879, he had published the result of his investigations, attributing caries of human teeth to a process of inflammation, a view which was opposed by many, on the ground that there was not sufficient living tissue in the dentine to permit of inflammation, even under the irritating process of caries. He said that the more he studied specimens of carious teeth, the more firmly he became convinced that his original position was correct. The recent article referred to clearly establishes the correctness of that position, even where only slight caries exists, and sometimes even when no caries at all is present.

Nothing of importance, either in Anatomy or Histology, had been brought to the notice of Section V. Only one paper was offered, which Dr. Abbott proceeded to read, under the title

HYPEROSTOSIS OF ROOTS OF TEETH.

Under this term the author includes all forms of pathological new growths of cementum, such as osteoma, exostosis, hypertrophy of the cementum, etc. As causes of this condition he enumerated irritation of the cementum through caries of the crown or neck, or exposure of the pulp; localized irritation of the pericementum, as from gout, etc., and irritation of the pericementum of upper teeth from gravitation, after-loss of antagonizing teeth.

Tumors have been considered as the result of chronic irritation of tissue. The theory of Cohnheim was that they were the result of a "misplacement of embryonal germs." The author of this paper thought that, on the one hand, such misplacement might occur without causing tumors, and that, on the other, tumors were found that were not even traceable to this cause.

Chronic irritation of the cementum may result in a new formation of cementum, but the question was, can it occur after the cementum has been fully formed? This question he answered in the negative, basing his opinion upon the microscopical studies of such formations, which had convinced him that it was, in most instances, a fœtal malformation.

If the roots of a carious tooth were found in an hyperplastic condition, it would at once be inferred that the inflamed pulp had led to pericementitis, and this to hyperostosis of the roots; but if all the roots were uniformly enlarged or fused together, it would be just as possible that this condition had existed before caries made its appearance, especially if, on grinding such teeth for microscopical examination, it was found that caries had not been sufficient to cause inflammation of the pulp, or if the condition of the dentine was such as could only have resulted from malformation at the beginning of its growth in fœtal life.

From a careful study of a large number of specimens, he felt convinced that the teeth were sound and the pulps alive when the bony growth was formed; in fact, he doubted the possibility of an osseous new formation after the death of the pulp; that if it had existed previously, its growth would cease with the life of the pulp. If a tooth was extracted to relieve pericementitis and found to be exostosed, it would hardly be considered that the pericementitis had caused the exostosis; the contrary would be the conclusion, the enlargement being the primary, and the pericementitis the secondary features. Cementum being identical with bone tissue, they are undoubtedly both built from medullary or embryonic tissue, whether cartilage or fibrous connection tissue is formed first. The lacunæ contain living protoplasm, and the canaliculi for tenants hold fibres of living matter. Only one portion of the protoplasm is transformed into basis-substance, that is, the lifeless liquid, which changes into a solid glue-yielding mass, forming the matrix into which is infiltrated the lime-salts. Augmentation of the cementum is impossible without a preceding augmentation of the medullary tissue, this being caused by increased nutrition or an irritation.

Bödecker has demonstrated that in normal cementum the lacunæ contain protoplasm, a portion of which is living matter, the whole basis-substance being traversed by a delicate reticulum containing threads of living matter, the cementum not being a mere inert mass of lime-salts with hollow lacunæ and canaliculi, even pathological cementum being endowed with the same properties of life. The process of the development, growth, enlargement, destruction and re-formation of cementum can be understood only as it is admitted that it is a living tissue.

The disease of hyperostosis attacks only bicuspids and molars, and more frequently the teeth of the upper jaw than the lower. These malformations cause facial neuralgia of the most severe and unyielding character, its diagnosis being at the same time very obscure. The symptoms are usually a slight continued uneasiness in the jaw, with a little soreness of the tooth in biting, increased by excessive pressure, the pain usually not localized positively in any one tooth. Sometimes several are extracted before the exostosed tooth is found and relief afforded. The pain, however, often continues, even after the last tooth is extracted. The different varieties of hyperostosis, as illustrated in over seventy teeth, were classified by the author under four heads:

(1st.) Circumscribed hyperostosis;

(2d.) Diffused hyperostosis, with roots separated;

(3d.) Diffused hyperostosis, with roots united;

(4th.) Two or more teeth united through hyperostosis.

These groups were again subdivided, the first into—

(*a.*) Osteoma on the body of the root;

(*b.*) On the apex of the root.

Each may vary in size from microscopic to the size of a lentil or pea, but usually with nodular surface. This form usually appears on teeth of which the crowns have been more or less destroyed by caries, with, probably, long exposed pulp, and is usually attributed to localized pericementitis. As, however, the death of the pulp stops the formation of osteoma on the roots, such tumors must have commenced long before the exposure of the pulp, causing a slight but constant irritation of that organ, and transferred to the pericementum, the irritation of the pericementum, while the pulp is living, causing the increased cementum.

In the second group, the roots are affected without the least symptom of disease upon the exposed portion of the tooth, the malformation probably dating from the beginning of the formation of the cementum. In these, the hyperostosis invades the roots from the apex to the middle, or two-thirds their length, or even to their necks, the bulging of the cementum sometimes overlapping the enamel, the roots looking clumsy and shortened, the mass partially filling the space between them.

In the third group the apices may be free and straight, or free and curved, or the roots may be united their entire length, the osseous outgrowth accumulating at the point of junction of the roots.

The third class of this group is quite common, this being almost the normal condition of wisdom teeth, only slight furrows indicating any separation of the roots, and the osteoma, when found, a clumsy, nodular mass.

The fourth group is of rather rare occurrence. The teeth may be united either at their apices or at their middle, or there may be a complete union of the roots. It is probable that at least one of the germs was malposed at the time of embryonic arrangement, and that the alveolar septum did not form at all, the mutual pressure from development of the roots causing the irritation which led to the new formation.

In the collection of teeth from which these studies were made, was one exception to the rule that only bicuspids and molars are affected by exostosis, the specimen referred to being a completely united upper canine and lateral incisor, with only shallow furrows reaching nearly to a common apex, with single large foramen—evidently a case of fœtal malformation.

Microscopical examination of sections of teeth affected with this disease showed the dentine in some cases normal, and in some the so-called interglobular spaces; in others the dentinal canaliculi of the root were arranged in bundles, between which were areas nearly or quite destitute of canaliculi, but with small interglobular spaces. The interzonal layer, between the dentine and cementum, exhibited osteo-dentine, the result of incomplete calcification, extending into the neck of the tooth, though less than on the roots. At the junction of the roots the cementum is found in connection with an irregular formation of dentine, or vaso-dentine, as illustrated by Wedl. Next to the layer of osteo-dentine is invariably a granular layer, which is destitute of cement corpuscles.

The hyperplastic cementum is often traversed by medullary canals, carrying central blood-vessels. The dentine often shows interglobular spaces filled with granular protoplasm serving as the termination of dentinal canaliculi, with their fibres of living matter.

Vaso-dentine presents to the naked eye a high degree of transparency, low powers of the microscope showing a varying number of medullary canals. The canaliculi contain medullary tissue and capillary blood-vessels, with glistening granules of lime-salts sometimes, in dilated portions of the canals. Sometimes extremely fine canaliculi run in fan-shaped groups, or they may assume the shape of a fountain, or other beautiful and striking figure. Higher powers of the microscope plainly show the medullary contents of the medullary canals, in which may be seen capillary blood-vessels. The canals and blood-vessels are unquestionably in communication with the blood-vessels of the pericementum.

In all of the specimens, the pulp chamber and often the canals appear considerably narrowed by formations of secondary dentine, the pulp-tissue containing "pulp-stones," or minute calcareous depositions. The enamel of such teeth is usually imperfectly calcified, with irregular and curly enamel rods.

A carefully decalcified portion of hyperplastic cementum, mounted in glycerine, under very high power, is found to be identical in structure with normal cementum. All canaliculi hold filaments of living matter, hyperplastic as well as normal cementum being a living tissue throughout. Between the basis-substance and the filaments of living matter, a slow circulation is going on, the liquid carrying nourishment and taking away the effete material.

Thus the pathological changes of a pathological tissue become intelligible, and cementitis of hyperplastic cementum understood.

The reading of this paper having taken till 10 P. M., discussion was postponed until the next session.

Adjourned to 9 A. M.

THURSDAY, AUGUST 5TH.

The meeting was called to order at 9.30 A. M., the President in the chair. The minutes of the previous session were read and approved.

By unanimous consent supplementary report was made from Sec. I, mentioning the processes and appliances of Dr. J. Rollo Knapp, of New Orleans. His porcelain-faced, or all gold crowns, admit of very little criticism, though requiring a skillful and careful manipulator to fabricate them. His very original blow-pipe, which utilizes compressed nitrous oxide with illuminating gas, produces a powerful oxy-hydrogen flame, and promises to be a most useful appliance for laboratory use.

Dr. Knapp being called upon to explain his methods, said that he could add nothing to what he had showed in his clinics. He would be glad to furnish all needful information to those desiring it.

Dr. N. W. Kingsley said it was pitiful to think of the time and efforts wasted in the old methods of producing the results which Dr. Knapp accomplished with such ease by such simple means. It pleased him to see his own pet methods carried to perfection. His method of producing a metallic die upon which to swage up his crowns is the most simple ever seen, while his compound blow-pipe does its work most beautifully.

Dr. Frank Abbott said he was completely carried away by the simplicity of his methods and the perfection of his results.

Section I was finally passed. The Committee on Credentials reported additional delegates present from the Vicksburg Dental Association, Wisconsin State Dental Association, Fifth District New York Dental Society, Alumni of Dental Department University of Pennsylvania, Northern Ohio Dental Society, Susquehanna Valley Dental Society, Kentucky State Dental Society, New York State Dental Society, Missouri Dental College.

Dr. Pierce, for the Committee on Voluntary Essays, reported two papers received. One had been returned as incomplete. The other was too long to be read as a whole, but the committee recommended that certain portions be referred to the Publication Committee.

*Prof. Taft*—Thought that if the paper was a good one, the Association should hear it. It was discouraging to persons who had devoted time and labor to the preparation of papers, to have them thus cast aside.

*Dr. Kingsleg*—Thought it not fair to mutilate a paper. If not accepted as a whole, it should be rejected as a whole, and returned to the author.

*Dr. Atkinson*—Said that the law of the society permitted the committee to do what they thought right with papers presented to them.

After some further discussion, the report of the committee was accepted.

On motion of Dr. Ingersoll, it was made the duty of the Executive Committee to provide a register for each annual meeting.

DISCUSSION OF SECTION V.—ANATOMY, HISTOLOGY AND MICROSCOPY.

*Dr. Atkinson*—Objected to the term hyperostosis as applied to the paper read, Hypercementosis being more appropriate to the conditions described. The illustrations he considered the very culmination of drawing. There was much in the paper that was worthy of attention, but there was also much that was mere fuss and feathers. Doctrines were pronounced as settled that were only hypothesis and assumption. It was only an assumption that the predisposition to hypercementosis existed from *in utero*, or that the work carried on was only during the life of the pulp.

It was potentially resident in the tooth-germ, and with new energies we get increased deposits of protoplasm, with deposits of lime-salts to make it visible as we see it. Much of the trouble is due to want of use. We should not ask why, but how?

The microscope reveals *how*, but only after the work is done. We find only the tracks; we don't know what has been there. We say arsenic causes certain results, but the arsenic is only the vehicle; it is the energy behind that causes. Mercury follows the same law; nutrition, also, is operated by the type behind it.

*Dr. Thompson*—Inquired if there might not be a nodular idiosyncrasy tending to produce such deposits on the bones and teeth?

*Dr. Atkinson*—Said he had yet to see the first well authenticated instance of hypertrophy or exostosis on a tooth which had never lost its occlusion.

*Dr. Wells*—Said he had had the proof in his own mouth, in a tooth which Dr. Fuller had extracted.

*Dr. Taft*—Knew of a case where antagonism was perfect and yet there was a large increase of cementum. He thought, however, that when an inferior antagonizing tooth was lost, the force of gravitation might cause an irritation that would create an abnormal growth of tissue.

*Dr. Morgan*—While accepting the statements made, thought the inferences incorrect. Any increase of cementum being due to function of the dental membrane, he did not see how it could be affected by the death of the pulp. The teeth being somewhat analogous to the other structures, as in general surgery there is healing by first intention, so where teeth are worn by attrition, in the efforts of nature to repair the loss of substance, filling in the tubuli with lime-salts, the increased sensitiveness of dentine may be due to the increased circulation of these reparative efforts, and not to inflammation.

*Dr. Ingersoll*—Said that confusion arose from a misconception of the term inflammation, and what it implies. All the phases of inflammation are not manifested in every tissue. Inflammation of cartilage is as genuine as inflammation of vascular tissue. It has been said that because the blood corpuscles cannot enter the tubuli there can be no inflammation, but that does not follow. Where there is nutrition there is circulation, and with vascular action there may be inflammation. If we accept the theory of inflammation of dentine, this becomes another factor in decay. Dentine and bone are constructed on much the same plan, and in both the medullary tissue may undergo inflammation, in bone the swelling being at the expense of the walls of the cancelli, with consequent softening of bone; in dentine the swelling of the dentinal fibrils is likewise at the expense of the hard tissues. In what we call demineralized dentine, the tubuli are broken down by the expansion of the fibrils.

*Dr. Abbott*—Said that his conclusions were merely his own opinions, based upon careful observations. He did not believe that increase of cementum could take place unless all the functions of the tooth were in working order. We find the extra growth on the roots of teeth, but we have no means of knowing when it begins. He hoped that enough interest had been excited to induce some one else to take up the work, and prove whether his conclusions were right or wrong.

The subject was passed.

SECTION VI.—PATHOLOGY, THERAPEUTICS AND MATERIA MEDICA.

Dr. Marshall read the report of this Section. They had not expended the $200.00 assigned them for scientific investigations, but had been engaged in maturing their plans, designing to use it, together with the appropriation for the present year, in the employment of recognized talent and ability, in making a special study of pyorrhœa alveolaris in all its phases—its deposits, exudations, secretions and microbes, with the characteristics of the saliva and urine, etc.

Dr. Hooper read a paper, giving the details of a case of extirpation of the inferior dental nerve. The patient had suffered for years with so-called facial neuralgia, and had had all the teeth extracted with only temporary relief. Since the operation, there had been no recurrence of the pain.

Dr. Hooper also exhibited a large piece of necrosed bone removed from the left inferior maxillary of a boy of twelve years of age, the result of an abscessed tooth which had been allowed to break on the outside, having been treated for nine months by physicians.

He also related the history of a case of supposed cancer, treated by several physicians, and lanced several times, arising simply from a dead pulp in the right central incisor. By proper treatment of the tooth the "cancer" was cured as well as persistent headaches, the deafness being also much improved.

Dr. A. W. Harlan read a paper entitled

BACTERIO-THERAPY.

This paper embodied the results of systematic experimentation in ascertaining the relative value of various antiseptics and disinfectants which, without such determination, are used empirically. The distinction between antiseptics, disinfectants, and germicides or microbicides, was drawn, and the conditions described, which indicate one or the other, or both.

Dr. McMillan, of Kansas City, presented an interesting pathological specimen, the skull of a negro, who had died at the age of fifty-five years, without having ever opened his mouth, having what was supposed to be congenitally anchylosed jaws. He had taken his food through the opening made by missing front teeth.

The subject was passed without discussion.

SECTION VII—PHYSIOLOGY AND ETIOLOGY.

Dr. Storer How read a paper on the practical value to the dentist of Litmus Tests of the Oral Fluids, as aids in determining the causes and cure of dental lesions, and in guiding the choice of filling materials. Through these studies new filling materials might be derived and new reagents discovered.

Dr. A. H. Thompson, Topeka, Kansas, read a paper on Protoplasmic Nutrition and Molecular Metamorphosis in the Dental Tissues.

He spoke of the mysterious powers of differentiation possessed by protoplasm, each tissue selecting from the nutrient stream the particular elements necessary to elaborate, whether nerve or muscle, bone or tooth-enamel, nutriment being carried in and waste carried out, each tissue performing its allotted work in the economy of the organism.

He said that, while it is a mooted question whether enamel should be classed as a vital or exfoliated product, the organic areas of protoplasmic elements which exists in it while the life of the pulp is maintained, prove it to be vital, though when the pulp perishes the enamel is as dead as hair or hoof cut off from nourishing currents. The pulp selects from the pabulum the organic and inorganic elements required to construct the different tissues of the tooth. The contents of the dentinal tubuli convey nutrition into and waste from its tissue. Throughout the enamel there are areas of living matter also, conveying limited nutrition with corresponding elimination of waste, by means of the connection with the living matter of the dentine at its periphery. This movement is maintained by osmosis, the hunger of the tissue causing it to draw in the pabulum; the more vital and vascular the tissues, the greater being the absorption of pabulum and consequent throwing off of waste. The dentinal tubuli anastomose with the canaliculi of the cementum, the circulation being thus continuous through the two tissues, maintaining some vitality in the dentine, even after the death of its life-source, the pulp. By similar anastomosis, between the dentinal tubuli and the areas of living matter in the enamel, nutrition is also conveyed to the latter tissue, though in less degree.

Molecular metamorphosis is therefore possible throughout the entire tooth structure and its tissues, subject to the pathological conditions of the circulating fluids. Increased density, or calcification in tooth structure, from eruption to adolescence, being admitted (as through the accelerated physiological activity of gestation and lactation), decalcification must also be admitted. In other conditions of ill-health we see teeth that were of good structure for years taking on a condition of softening, something acting through the circulating fluids, disintegrating and carrying off the lime-salts. The bones lose their phosphate of lime, and the brain loses its phosphorus, until a phosphoric famine begins all over the body. Sometimes construction is held in abeyance while waste goes on; or again, construction goes on while waste is lessened. We can observe this in the dentine, and it probably takes place in the enamel also. Where removal of waste in the teeth is not followed by compensating reconstruction, softening of the tissues will result, with loss of resistance to caries. In certain inflammatory conditions the removal of elements is followed by molecular synthesis, the basis-substance as well as the calcific elements being reproduced. The mystery of this molecular work is impenetrable.

Adjourned to 8 P. M.

(TO BE CONTINUED.)

